# Performance Comparison of Deep Learning Autoencoders for Cancer Subtype Detection Using Multi-Omics Data

**DOI:** 10.3390/cancers13092013

**Published:** 2021-04-22

**Authors:** Edian F. Franco, Pratip Rana, Aline Cruz, Víctor V. Calderón, Vasco Azevedo, Rommel T. J. Ramos, Preetam Ghosh

**Affiliations:** 1 Institute of Biological Sciences, Federal University of Para, Belem, PA 66075-110, Brazil; edianfranco@ufpa.br; 2 Laboratory of Virology and Environmental Genomics, Instituto de Innovacion en Biotecnologia e Industria (IIBI), Santo Domingo 10104, Dominican Republic; 3 Instituto Tecnológico de Santo Domingo (INTEC), Santo Domingo 10602, Dominican Republic; victor.calderon@intec.edu.do; 4 Department of Computer Science, Virginia Commonwealth University, Richmond, VA 23284, USA; ranap@vcu.edu (P.R.); pghosh@vcu.edu (P.G.); 5 Programa de Pós-Graduação em Enfermagem, Federal University of Para, Belem, PA 66075-110, Brazil; alinecruz@ufpa.br; 6 Institute of Biological Science, Federal University of Minas Gerais, Belo Horizonte, MG 31270-901, Brazil; vasco@icb.ufmg.br

**Keywords:** cancer subtype detection, multi-omics data, data integration, autoencoder, survival analysis

## Abstract

**Simple Summary:**

Here, we compared the performance of four different autoencoders: (a) vanilla, (b) sparse, (c) denoising, and (d) variational for subtype detection on four cancer types: Glioblastoma multiforme, Colon Adenocarcinoma, Kidney renal clear cell carcinoma, and Breast invasive carcinoma. Multiview dataset comprising gene expression, DNA methylation, and miRNA expression from TCGA is fed into an autoencoder to get a compressed nonlinear representation. Then the clustering technique was applied on that compressed representation to reveal the subtype of cancer. Though different autoencoders’ performance varies on different datasets, they performed much better than standard data fusion techniques such as PCA, kernel PCA, and sparse PCA.

**Abstract:**

A heterogeneous disease such as cancer is activated through multiple pathways and different perturbations. Depending upon the activated pathway(s), the survival of the patients varies significantly and shows different efficacy to various drugs. Therefore, cancer subtype detection using genomics level data is a significant research problem. Subtype detection is often a complex problem, and in most cases, needs multi-omics data fusion to achieve accurate subtyping. Different data fusion and subtyping approaches have been proposed over the years, such as kernel-based fusion, matrix factorization, and deep learning autoencoders. In this paper, we compared the performance of different deep learning autoencoders for cancer subtype detection. We performed cancer subtype detection on four different cancer types from The Cancer Genome Atlas (TCGA) datasets using four autoencoder implementations. We also predicted the optimal number of subtypes in a cancer type using the silhouette score and found that the detected subtypes exhibit significant differences in survival profiles. Furthermore, we compared the effect of feature selection and similarity measures for subtype detection. For further evaluation, we used the Glioblastoma multiforme (GBM) dataset and identified the differentially expressed genes in each of the subtypes. The results obtained are consistent with other genomic studies and can be corroborated with the involved pathways and biological functions. Thus, it shows that the results from the autoencoders, obtained through the interaction of different datatypes of cancer, can be used for the prediction and characterization of patient subgroups and survival profiles.

## 1. Introduction

Due to technological advancement and decreasing costs, high-throughput sequencing technology such as RNA-seq, SNP-chip, UPLC-MS, and GC-MS techniques generate extensive and diverse amounts of omics data that allow biologists to understand the different processes and interactions within biological organisms with unprecedented detail. These omics technologies provide the ability to interpret and explain the genome through DNA sequencing, genome expression based transcriptome studies, protein identification from the proteome, and others. However, such individual data can only provide limited information on the molecular complexity occurring inside the organisms due to the multi-level regulation inside biological units [1]. For example, we observe the combined effects of transcripts and methylome in the tumor cell due to genomics defect [2]. Considering gene expression data alone ignores the effect of point mutation, which alters the efficacy of gene products [3]. Furthermore, the dimension and the diversity of such data make it extremely challenging to perform proper data handling and in-depth analysis. Hence, there is an urgent requirement for mathematical models that can efficiently fuse these diverse molecular data from different measurements and provide us with a comprehensive and robust insight into biological phenotypes.

**Figure 1:**
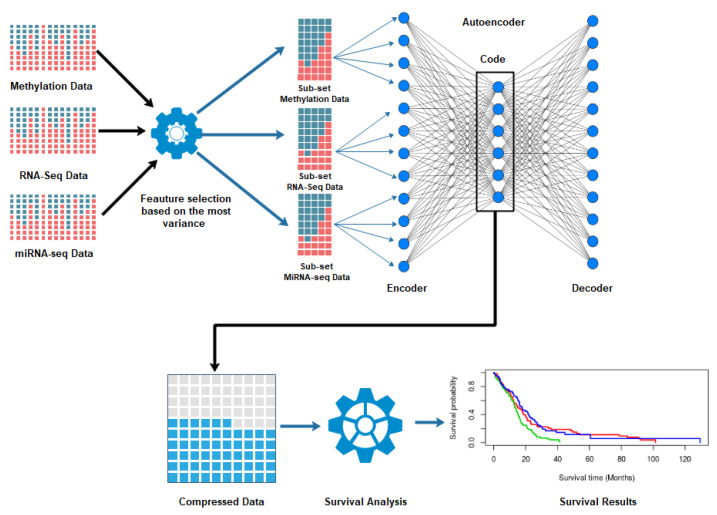
The workflow of subtype detection using autoencoders. First, we perform feature selection from the multi-omics data of the same patients from the TCGA database. Next, autoencoders fuse the selected features by encoding and decoding. Then, we run two clustering algorithms on the patient similarity networks constructed from the bottleneck layer to identify the subtypes of cancer. Finally, we run a survival analysis of the identified clusters to validate the results.

Ritchie et al. [4] define multi-omics data integration as the method in which diverse types of omics data are combined as predictor variables to allow more accurate and extensive modeling of complex traits or phenotypes. The integrated multi-omics methods permit the identification of crucial genomic factors and biomarkers, generate models to explain and predict disease risk, and understand the genetics and genomics architecture of complex phenotypes. Such integrated data also provide a holistic view of the biological system compared with traditional data-based methods [4,5,6,7]. Several data fusion models have been proposed recently, which fall into the following three categories: (a) early fusion, (b) intermediate fusion, and (c) late fusion. One example of a data fusion algorithm is similarity network fusion (SNF) [8]. Here, diverse types of data are first normalized into a network form through a nonlinear kernel function. Next, SNF efficiently fuses these networks through an iterative fusion algorithm. Recently, the deep-learning framework of autoencoders also exhibited significant potential as a data fusion algorithm. An autoencoder reconstructs its input by a nonlinear transformation of its original input features. Hence, in this process, the autoencoder generates new nonlinear features from its original input feature-set. Several cancer studies used autoencoders to analyze multi-omics data. Autoencoder based data integration has also been successfully applied to predict drug response [9] and kidney graft survival analysis [10]. The autoencoder is an unsupervised deep learning (DL) algorithm for dimensionality reduction and heterogeneous data integration based on feed-forward neural networks [11]. Autoencoders can automatically learn nonlinear features from the unlabeled data after setting the output value equal to the input value. An autoencoder is constructed by combining simple neurons where the output of one layer of neurons acts as the input to other layer of neurons. The autoencoder network forms a “butterfly” structure, where the number of inputs is equal to the number of outputs and consists of bottleneck hidden layers in the middle. This design drives the network to seek a compressed representation of the data while preserving the input data’s most important features (Figure 1). The architecture of an autoencoder allows it to concatenate the features and information of different omics sources [12,13,14,15].

A critical application of such data fusion algorithms is cancer subtype detection using omics data. Multiple oncogenes are involved in a heterogeneous disease like cancer, and they are perturbed through several pathways. Cancer patients’ severity and their survival also differ considerably depending upon this perturbation. For example, Glioblastoma multiforme (GBM) has four established subtypes: Classical, Mesenchymal, Neural, and Proneural. Subtype detection is a complex problem and frequently requires the fusion of various heterogeneous datasets. Recently autoencoders are also used for subtype detection problems for Liver cancer by fusing three heterogeneous data types. For example, Chaudhary et al. [16] used autoencoders on methylation, RNA-seq, and miRNA-Seq data from liver cancer patients to develop a robust model to predict two distinct survival groups. Also, Tan et al. [17] used the denoising autoencoder to develop a model that can identify and extract an intricate pattern from omics data in breast cancer. Deep learning autoencoders were also used for subtype classification in colorectal cancer using multi-omics data [18], while [14] applied autoencoders to identify two subtypes in neuroblastoma.

In Zhang et al. [19], the authors used a variational autoencoder to integrate multi-omic cancer data. The model was used to develop pan-cancer classification analysis and obtained an average precision of 97.49% after 10-fold cross-validation of 33 tumor types and normal samples. Simidjievski et al. [20] explored the different architectures, designs, and construction of multi-omic data integration methods using Variational Autoencoders; they demonstrated that autoencoders are suitable methods for representing data and the production of stable and accurate diagnostics. To study the genes that mediate human lung adenocarcinoma, a model was created based on the denoising autoencoder. This allowed the identification of more positive genes related to this type of cancer than other methods [21].

Depending upon the deep learning layer construction and regularization, an autoencoder can be of different types such as vanilla autoencoder, denoising autoencoder, sparse autoencoder, and variational autoencoder. Though autoencoders showed promise for data fusion and subtype detection in the recent past, the performance of different types of autoencoders on the different datasets is still unknown. In this work, we compared the performance of four different autoencoders to integrate and reduce multi-omics data. By data fusion, autoencoders created new features to represent the input datasets. The new features were used to implement a survival-based clustering algorithm to define groups of patients with a similar distribution of features and survival prognosis. We evaluated the efficiency of the different autoencoders (vanilla, denoising, sparse and variational) for the fusion and reduction of cancer data dimensions from different sources such as RNA-seq, methylation, and miRNA-Seq, on four different cancer types.

## 2. Materials and Methods

### 2.1. Dataset and Prepossessing

We obtained the multi-omics cancer data from The Cancer Genome Atlas Program (TCGA) database. TCGA consists of more than 20,000 primary cancer samples over 33 cancer types. We applied autoencoder-based subtyping on datasets of four cancer types: Glioblastoma multiforme (GBM) and Colon Adenocarcinoma (COAD) from TCGA and Kidney renal clear cell carcinoma (KRCC) and Breast invasive carcinoma (BIC) from TCGA but preprocessed by Wang et al. [8]. We utilized three types of data: gene expression, DNA methylation, and miRNA expression.

GBM is one of the most aggressive brain tumors; the survival estimate of a diagnosed patient is 13 months on average, even after chemotherapy and radiotherapy treatments. We analyzed data collected from 276 patients of this cancer type (male—164, female—112), with 17,814 features for mRNA expression, 470 features for miRNA expression, and 13,000 features for DNA methylation. BIC is one of the most common types of breast cancer and about 80% of breast cancers are invasive [22]. From BIC dataset [8], we analyzed the data collected from 106 patients, with 335 features for the miRNA expression, 23,094 features for DNA methylation, and 17,814 for mRNA gene expression. COAD is a type of cancer that usually arises from the epithelium lining inside the large intestine. This type of cancer is more prevalent in the population aged over 50 and in countries with a low fiber diet, such as Europe, the USA, and Australia. COAD dataset represents approximately 10% of diagnosed cancers [23,24]. From the COAD dataset, we analyzed data collected from 92 patients with 17,814 features for mRNA expression, 23,087 features for DNA methylation, and 311 features for miRNA expression. KRCC is the most common type of kidney cancer and affects the lining cell and tiny tubules that filter waste from the blood and produce urine in the kidney. This type of cancer is more prevalent in men over 55 years of age [25,26]. From this dataset, we analyzed data collected from 122 patients, with 17,898 features for mRNA expression, 24,959 features for DNA methylation, and 329 features for miRNA expression.

First, we downloaded the TCGA dataset comprising gene expression, DNA methylation, and miRNA expression from the TCGA database using the TCGAbiolink package [27]. Then, we chose the common patients in these datasets for our analysis and also downloaded the patients’ clinical data to perform survival analysis. Next, we scaled each data using the following equation.

(1) $$X_{n}=\frac{X_{i}-x_{min}}{x_{max}-x_{min}}$$

where $X_{i}$ is the data instance while $x_{max}$ and $x_{min}$ are the minimum and maximum absolute value of feature *X* respectively, and $X_{n}$ is the feature after normalization. We chose 100/400/500 number of important features from each dataset based on maximum variance (VAR) using the function FSbyVar from the CancerSubtypes package in R [28] as shown in Figure 1. However, other robust variable selection techniques [29] can also be used to select relevant and robust features; we did not implement these other methods as our goal in this paper was to primarily assess the performance of autoencoders for data fusion. These selected features were fed into the autoencoders as the input.

### 2.2. Autoencoder Construction

**Figure 2:**
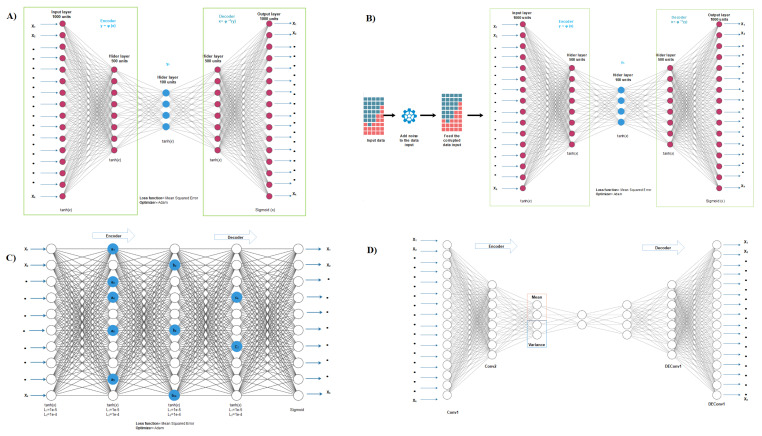
Autoencoder configuration: (**A**) Vanilla autoencoder; (**B**) Denoising autoencoder; (**C**) Sparse autoencoder, and (**D**) Variational autoencoder.

An autoencoder can be of different types based on its construction as shown in Figure 2. One simple form of an autoencoder is vanilla autoencoder, traditionally constructed with a single layer of encoder and decoder. The learning minimizes the following loss function.

(2) L(x,g(f(x)))

where *L* is the loss function of input *x* and output g(f(x)). Due to the nonlinearity of the encoder and decoder’s activation function, the vanilla encoder learns nonlinear features from the data. This is not feasible from the linear feature deduction methods such as Principal Component Analysis (PCA) [30]. A vanilla autoencoder with multiple hidden layers is called a deep vanilla autoencoder.

Though vanilla autoencoder is simple, there is a high possibility of over-fitting. Denoising autoencoder, sparse autoencoder, and variational autoencoder are regularized versions of the vanilla autoencoder. Denoising autoencoder reconstructs the original input from a corrupt copy of an input; hence, it minimizes the following loss function.

(3) $$L(x,g\left(f\left(\tilde{x}\right)\right))$$

where *L* is the loss function of input *x* and output $g(f\left(\tilde{x})\right)$. A corrupt copy of input is formed by introducing noise to the original input. Denoising is achieved through stochastic mapping by setting some input values to zero. The added noise helps the autoencoder learn features other than the original features directly from the data.

Sparse autoencoder is a regularized version of vanilla autoencoder with a sparsity penalty Ω(h) added to the bottleneck layer. The learning of a sparse autoencoder minimizes the following loss function.

(4) L(x,g(f(x)))+Ω(h)

The sparsity penalty Ω(h) helps to learn the important features of data even when there are many hidden units in the autoencoder.

Variational autoencoder uses a strong assumption about latent variables by generally using a latent Gaussian distribution [31,32]. It imposes a constraint in the encoder network, which forces the bottleneck layer to follow a Gaussian distribution. The learning of a variational autoencoder minimizes the following loss function

(5) L(x,g(f(x)))+L(l)

where L(l) is the latent loss, measured in terms of the Kullback-Leibler divergence of the bottleneck layer to a unit Gaussian distribution, which quantifies the difference between them. This assumption generates the latent variable with a generalization of the network.

### 2.3. Autoencoder Implementation

We used the Keras library [33] with TensorFlow [34] background to implement the four distinct autoencoders compared in this paper. The autoencoders were trained on a Quadro P4000 GPU with 8 Gb RAM. For subtyping and survival analysis, we applied the CancerSubtype R package [28].

For the vanilla, denoising, and sparse autoencoders, we set 500, 100, 500 nodes respectively for the three hidden layers and 1000 nodes for both input and output layers. The number of nodes for the input and output layers, were selected based on the maximum variance of three data types as we selected 500 features from gene expression, 400 features from DNA methylation, and 100 features for miRNA expression. For the denoising autoencoder, we applied a noise factor of 0.5 in the input data network. For the sparse autoencoder, we set an L1 regularization penalty of 0.01 and an L2 regularization penalty of 0.01 on the nodes to induce sparsity. For the variational autoencoder we set four hidden layers with 1000, 500, 250 and 100 nodes respectively. Also, we used the sequential model for the decoder and the functional model for the encoder. We used the log variance and lambda layer to convert the standard deviation for numerical stability when necessary.

To optimize all the autoencoders we utilized an extension to the stochastic gradient descent (adam) algorithm [35]. For vanilla, sparse, and denoising autoencoders, we applied hyperbolic tangent (tanh) activation function on the input and hidden layers and sigmoid on the output layer. For the variational autoencoder, we applied a rectified linear activation function (ReLU) on the input and hidden layers and sigmoid in the output layer. Also, to measure the loss between the input layers (*X*) and the output layer ($X^{\prime }$), we chose the mean square error function for the vanilla and denoising autoencoders and the binary cross-entropy function for sparse autoencoder, and the negative log-likelihood function for variational autoencoder.

### 2.4. Clustering and Subtyping

The autoencoder transforms multidimensional features to a reduced number of features in the bottleneck layer. On this reduced feature set, we applied the standard subtyping method to subtype patients. First, we calculated the similarity of each patient pair considering these reduced set of features. Here, we used Euclidean distance and Spearman correlation as a similarity measure between two patients. Then, we employed an unsupervised clustering algorithm to cluster similar groups of patients. Here, we used an unsupervised subtypes discovery method combined with k-means [36] and Partitioning around medoids (PAM) [37] as our clustering methods. We executed the two algorithms (k-means and PAM) in a window between 3 and 6 clusters.

### 2.5. Evaluation Metrics for Subtyping

We utilized two different metrics to evaluate the performance of different autoencoders on the TCGA dataset. First, we performed survival analysis to evaluate the survival patterns from different subtypes. Next, we calculated the *p*-value of the log-rank test to identify the difference in Kaplan-Meier survival curves between different subtypes. Here, low *p*-value (<0.05) ensure high confidence of different survival times for the different identified subtypes.

We also used the silhouette width of the clusters to benchmark the performance of Clustering. Silhouette scores measure how well a patient is matched to its identified cluster compared to other clusters, i.e., inside the group versus outside the group. A high Silhouette value indicates a proper group distribution.

### 2.6. COX Model for Feature Selection

To validate the data fusion, we selected the two datasets (COAD and KRCC) that obtained the lowest results with the feature selection by the variance and made a new selection of features based on the COX proportional hazards model [38]. COX proportional hazards model is a regression model that predicts the relationship between the predictor variable and patients’ survival. Using the univariate COX model with a cutoff of *p* < 0.05, we selected 8788 features from the mRNA data, 400 features from DNA methylation data, and 16 features from the miRNA expression data from COAD datasets. Also, we selected 565 features from mRNA data, 419 features from DNA methylation data and 33 features from miRNA expression data. Next, we fed these selected features as input for vanilla, sparse, denoising, and variational autoencoder implementations.

### 2.7. Comparison with Other Data Integration Methods

We compare our results with other data fusion methods such as SNF, principal component analysis (PCA), kernel PCA and sparse PCA [39]. SNF is a computational method for the fusion of similarity network to aggregate multi-omics data [8]. In this method, we used the methylation and mRNA from GBM datasets. Before applying SNF, we performed a feature selection using the COX regression model. We selected 2806 features from the DNA methylation data and 3309 from the mRNA expression data. The SNF algorithm and the survival analysis were implemented with clusters from 3 to 6 using the CancerSubtype package.

PCA allows linear dimensionality reduction to project the data in lower-dimensional spaces. Whereas, kernel PCA is a nonlinear version of PCA and sparse PCA is a regularized version of PCA. We implemented PCA, kernel PCA and sparse PCA in Python using the sklearn package, and the features were selected based on the variance (0.90) in the GBM dataset. We used the PCA-transformed dataset as the input to the k-means/PAM Clustering algorithm for cancer subtype identification using the CancerSubtype package.

### 2.8. Differential Expression and Enrichment Analysis on Detected Subtypes

Lastly, we performed a differential expression (DE) and functional enrichment analysis of the clusters and compared the DE genes and enriched processes among the clusters. The DE genes were detected using the linear method LIMMA [40], while the functional enrichment analysis was performed using the ClusterProfiler [41] package in R. This can identify the critical genes that belong to a subtype and identify the functional processes which may lead to this outcome.

To explore the organization of the clusters, we performed a differential expression analysis using the GBM dataset. For the analysis, we downloaded the gene expression data for each cluster obtained from the different types of autoencoders and used the clustering algorithms (PAM and k-means) from the HT_HG-U133A platform, using the GDCquery, GDCdownload, and GDCprepare functions. Samples with Primary Tumor and samples with solid tissue normal were compared to get differential expression utilizing the TCGAanalyze_DEA function with fdr.cut=0.01 and logFC.cut=1.

For the enrichment analysis of the gene sets, we used the TCGAanalyze_EAcomplet function that allows us to obtain the biological processes, cellular components, and molecular functions of Gene Ontology (GO) [42], in addition to the enrichment of the pathways.

## 3. Results and Discussion

### 3.1. Performance of Different Autoencoders

We ran the survival analysis for 3 to 6 clusters for each autoencoder (Table 1 and Table 2). We noticed that the silhouette score differs depending upon the regularization methods. Hence, we chose the optimal cluster number for a disease based on counting the number of autoencoders that achieved a high silhouette score (>0.80). Next, we performed a log-rank test to check if the identified clusters have different survival profiles. The lowest *p*-values with a high silhouette score (>0.8) for the optimal cluster number were considered as the final cluster prediction. The performance of different autoencoders varies depending upon the dataset, and clearly there is no single winner architecture.

### 3.2. Performance of Different Autoencoders for Gbm

**Figure 3:**
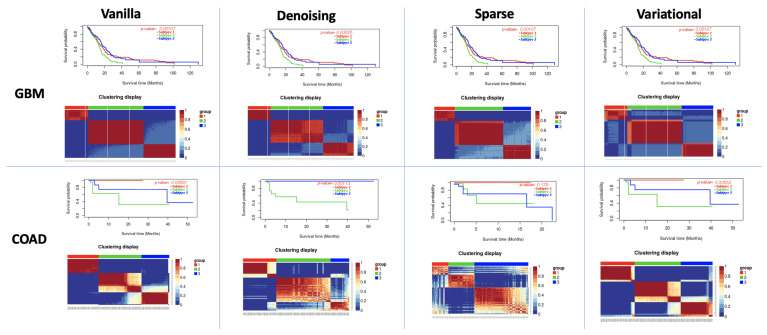
K-means survival analysis on Datasets. In each subfigure, (**Top**): Kaplan-Meier survival curves of three identified clusters. The log-rank test confirmed a difference in survival profiles among clusters; (**Down**): Patient to patient similarity and identified clusters on the dataset.

**Figure 4:**
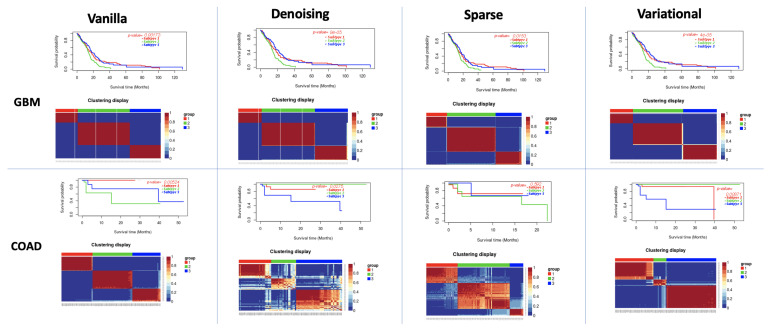
PAM survival analysis on Datasets. In each subfigure, (**Top**): Kaplan-Meier survival curves of three identified clusters; (**Down**): Patient to patient similarity and identified clusters on the dataset.

GBM is the most studied cancer for subtype detection using multiview learning. However, a different number of subtypes has been detected by different computational methods on different datasets (Figure 3 and Figure 4). Authors in [8] discovered three subtypes from 215 patients from TCGA using mRNA, miRNA, and DNA methylation data. While [43] classified GBM into the following four subtypes: (a) Classical, (b) Mesenchymal, (c) Neural and (d) Proneural. The authors in [44] also found three subtypes for the GBM dataset. We predicted three as the optimal cluster number. All eight autoencoders achieved a high silhouette score (>0.8), while the variational autoencoder with PAM/Spearman achieved the lowest *p*-value in the log-rank test.

### 3.3. Performance of Different Autoencoders for Coad

For COAD, based on the count of silhouette score cutoff, we predicted the optimum number of clusters as three (Figure 3 and Figure 4 and (Supplementary Materials S1). Four different autoencoders (Vanilla and variational autoencoders) achieved a high silhouette score for three clusters. The vanilla autoencoder with PAM/Spearman achieved the highest silhouette score of 0.96. We also observed a significant difference in the survival profiles between these clusters p=0.05. Moreover, all other autoencoders also detected a difference in survival time for K=3. It should be noted here that Wang et al. [8] also found three clusters in COAD based on the Eigen distance.

### 3.4. Effect of Different Similarity Measures

Calculating patient-to-patient similarity measure is a crucial step in subtype detection. We can use various similarity measures for subtype detection, in which performance can vary depending on the dataset. Here we observed that PAM clustering with Spearman distance usually performed favorably than the k-means clustering with Euclidean distance. PAM with Spearman achieved better clustering based on the silhouette score. However, the identified clusters using k-means/Euclidean distance commonly showed a lower *p*-value for the survival difference between the identified clusters.

### 3.5. Effect of Supervised Feature Selection

For the KRCC and COAD datasets, there was no significant difference in survival profile between clusters for most autoencoders. Hence, we chose a supervised feature selection algorithm COX to select the input features. The COX model is a supervised model that selects the genes based on the survival status of patients. We observed a significant improvement of the *p* value for survival difference between the clusters using this method (Table 3). However, we noticed a decrease in silhouette score than the VAR feature reduction method. Based on the silhouette score cutoff, the variational autoencoder with Spearman distance performed best, and the number of the chosen optimal clusters was 3. It identified 3 different clusters with significant survival difference *p* = 1 × 10${}^{-8}$. Also, for KRCC, the variational autoencoders achieved the highest silhouette score with three clusters. It also revealed a significant difference in survival profile between clusters.

### 3.6. Comparison with Other Subtype Detection Methods

**Figure 5:**
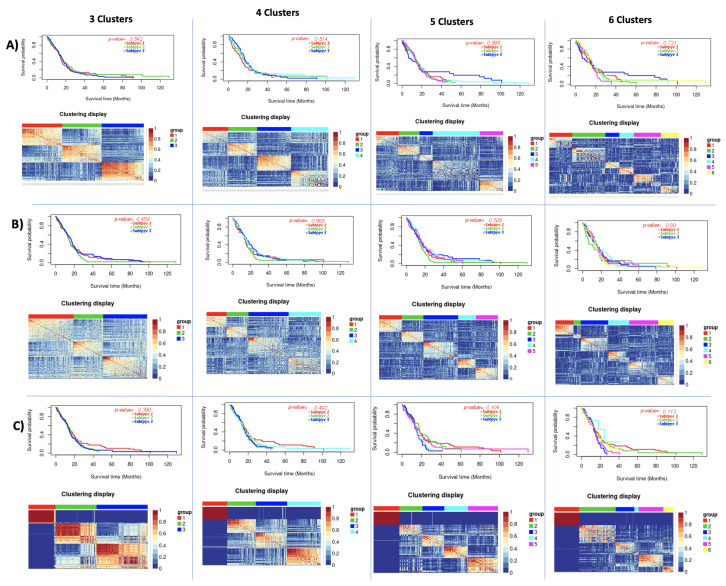
Principal component analysis results: (**A**) Principal Component Analysis (PCA) Results; (**B**) Kernel Principal Component Analysis (KPCA) Results; and (**C**) Sparce Principal Component Analysis (SPCA). Results In each subfigure, (**Top**): Kaplan-Meier survival curves of the identified clusters. (**Down**): Patient to patient similarity and identified clusters on the dataset.

**Figure 6:**
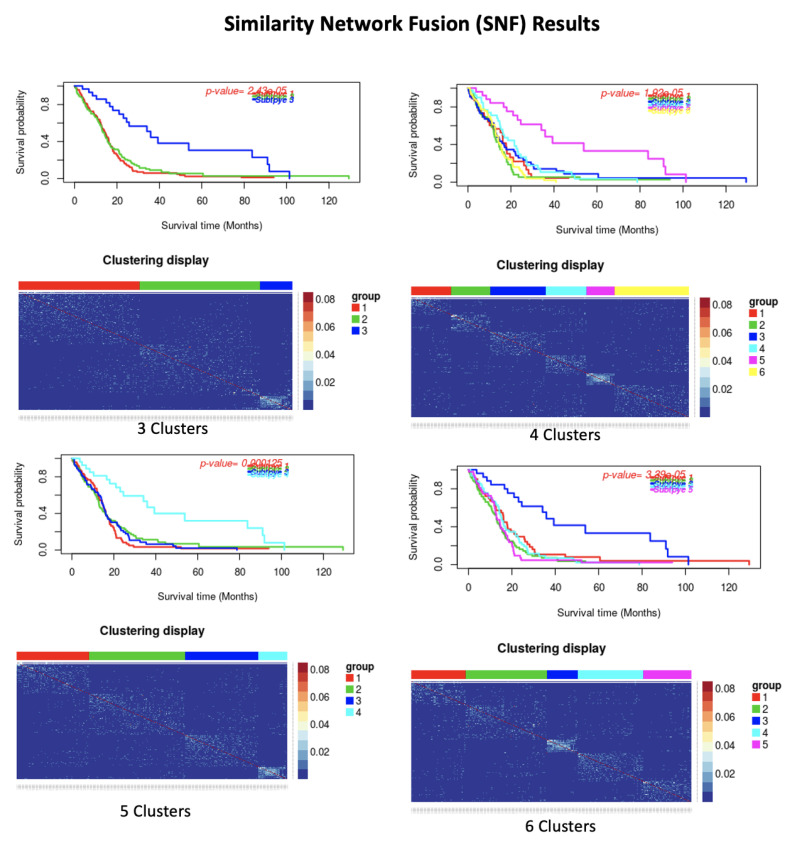
Similarity Network Fusion (SNF) results. In each subfigure, (**Top**): Kaplan-Meier survival curves of the identified clusters. (**Down**): Patient to patient similarity and identified clusters on the dataset.

Next, we compared the autoencoder subtype detection result with four other commonly used data fusion techniques: PCA, kernel PCA and sparse PCA and SNF (Table 4). PCA is a commonly used method for dimensionality reduction. Unfortunately, PCA performed poorly for subtype detection. The clusters identified by PCA using Spearman correlation did not significantly differ in survival time (Figure 5). SNF is another popular approach for data fusion. SNF showed comparable performance to autoencoders for subtype detection (Figure 6). However, SNF has a few additional hyperparameters, and the result is sensitive to hyperparameter selection.

### 3.7. Differential Expression and Enrichment Analysis on Detected Subtypes

**Figure 7:**
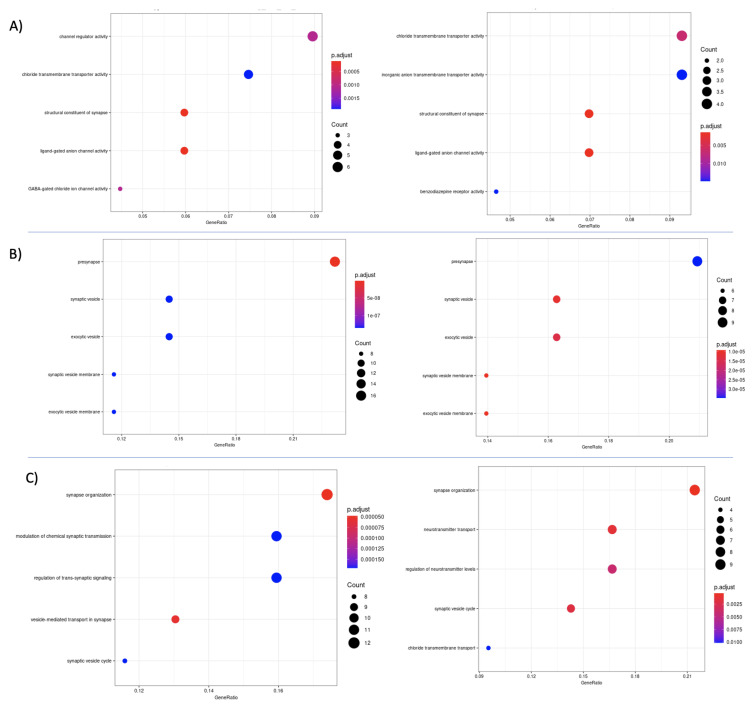
GO analysis was performed on the differentially expressed genes identified in the Denoising and Sparse autoencoders’ results in patients cluster 1 using the k-means algorithm. The GO results were used to analyze the (**A**) molecular functions, (**B**) Cellular components, and (**C**) Biological processes, identified by the Denoising Autoencoder and the Sparse Autoencoder data.

The Gene ontology (GO) and KEGG pathways’ enrichment showed numerous differentially expressed genes between GBM and control samples on the four autoencoders (vanilla, denoising, Sparse, variational) identified subgroups. The genes were related to cellular components, biological processes, and molecular function as shown in Figure 7 (and Supplementary Materials S2), which is similar to previous studies [45,46]. Some selection criteria were applied to increase the reliability and precision of the results as follows: (i) *p*-value < 0.05, (ii) reads count ≥6 (0 to 12), (iii) shared in the results from all autoencoders, and (iv) belong to at least two clusters.

First, we found that only synaptic organization is present among the three clusters (CL1, CL2, and CL3). According to the GO, the cell function called synaptic organization is a process that results in the assembly, an arrangement of constituent parts or disassembly of a synapse, the junction between a neuron and a target (neuron, muscle, or secretory cell).

Immune synapse occurs when a conjugate of T cells and their targets are formed and triggers the reorganization of surface receptors. Then actin accumulates at the contact site, forming the peripheral ring that delivers cytotoxic granules to the cytolytic synapse. The authors in [47] showed that impaired synaptic organization affects cell adhesion in T cells.

Second, we identified pre-synaptic and vesicle-mediated transport in cellular synapse components in at least two out of the three clusters we evaluated. These findings were similar to the study by Xiong et al. [48] when analyzing targets of genes differentially expressed in GBM samples from in silico analysis using the Gene Expression Omnibus (GEO) database.

A pre-synaptic terminal in a synapse secretes neurotransmitters and the postsynaptic terminal receives the neurotransmitters in its receptors [49]. This process is orchestrated by multiple and complex signaling pathways that differentiate the excitatory from the inhibitory pre-synapse; however, this process is still mostly unknown [50].

Yool et al. [51] identified that SYN1 (considered as a pre-synaptic marker) is expressed outside neural tissues that can mimic neurotransmission. Furthermore, glutamate self-stimulation in malignant cells favors proliferation, motility, excitotoxic cell death, and seizures in peritumor brain tissues [52]. Therefore, pre-synaptic hyper-expression is unfavorable to a good prognosis.

Vesicles have been extensively investigated as a repository and as a transportation mode of proteins, RNAs, and lipids between local and distant cells [53]. Vesicle-mediated intercellular communication, also known as surrounding tumor microenvironment (TME) is composed of malignant, benign cells and non-cellular components. It can interfere with gene expression by favoring a pro-tumorigenic microenvironment that modulates tumor behavior, aggressiveness, recurrence, and progression [54,55]. In GBM, the TME plays a crucial role in the progression of the GBM, with the vesicles being identified in the bidirectional communication between the tumor and the TME, in addition to favoring avoidance of apoptosis and therapeutic resistance [56], and also unfavorable to a good prognosis.

## 4. Conclusions

Recently, deep learning autoencoders are showing huge promise for multiview data fusion and cancer subtype detection. Here, we compared four regularized autoencoders for subtype detection for four cancer types from the TCGA database. Though the performance of different autoencoders varied on different datasets, in general vanilla and variational autoencoders showed the best performance to detect the subtypes. We also observed that PAM/Spearman similarity showed better performance than k-means/Euclidean clustering. We predicted the optimum number of subtypes for four cancer types by comparing the four autoencoders’ results. Moreover, DE analysis of the identified subtypes discovered critical genes and pathways in each subtype. Overall, we showed that multi-omics data fusion combined with subtype detection as proposed here can improve cancer patient care.

## Figures and Tables

**Table 1:** *p*-value of survival analysis results for the clusters generated with the autoencoder output.

Dataset	Number of Cluster	Autoencoder Vanilla		Autoencoder Denoising		Autoencoder Sparse		Autoencoder Variational	
		PAM/Spearman	k-Means/Euclidean	PAM/Spearman	k-Means/Euclidean	PAM/Spearman	k-Means/Euclidean	PAM/Spearman	k-Means/Euclidean
GBM	3	0.002	0.001	9 × 10${}^{-5}$	9 × 10${}^{-4}$	0.015	0.001	5 × 10${}^{-5}$	0.001
	4	0.002	2 × 10${}^{-4}$	0.06	2 × 10${}^{-5}$	0.109	6 × 10${}^{-5}$	0.006	6 × 10${}^{-5}$
	5	2 × 10${}^{-4}$	1 × 10${}^{-4}$	0.001	1 × 10${}^{-5}$	0.015	7 × 10${}^{-5}$	5 × 10${}^{-5}$	3 × 10${}^{-5}$
	6	3 × 10${}^{-4}$	2 × 10${}^{-5}$	0.003	4 × 10${}^{-5}$	0.018	1 × 10${}^{-5}$	1 × 10${}^{-4}$	2 × 10${}^{-5}$
BIC	3	0.0667	0.664	0.193	0.508	0.089	0.078	0.271	0.443
	4	0.0049	0.183	0.145	0.0275	0.016	0.304	0.0659	0.194
	5	0.322	0.0273	0.0481	0.0476	0.003	0.37	0.103	0.219
	6	0.212	0.621	0.0306	0.0457	0.007	0.0012	0.367	0.441
COAD	3	0.00524	0.00581	0.0275	0.00011	0.592	0.178	0.00871	0.0053
	4	0.0144	0.0135	0.044	0.0007	0.007	0.221	0.054	0.0181
	5	0.0309	0.031	0.0159	0.0041	0.0094	0.292	0.0951	0.0006
	6	0.0241	0.0336	0.0341	0.00547	0.97	0.212	0.0802	0.014
KRCC	3	0.288	0.392	0.165	0.135	0.346	0.229	0.00608	0.0266
	4	0.471	0.6144	0.437	0.47	0.614	0.174	0.0353	0.0393
	5	0.665	0.347	0.691	0.036	0.508	0.321	0.131	0.0141
	6	0.369	0.527	0.268	0.068	0.541	0.349	0.0669	0.0324

**Table 2:** Silhouette index results for the clusters generated with the autoencoder output.

Dataset	Number of Cluster	Autoencoder Vanilla		Autoencoder Denoising		Autoencoder Sparse		Autoencoder Variational	
		PAM/Spearman	k-Means/Euclidean	PAM/Spearman	k-Means/Euclidean	PAM/Spearman	k-Means/Euclidean	PAM/Spearman	k-Means/Euclidean
GBM	3	1	0.91	0.98	0.91	0.97	0.83	0.98	0.87
	4	0.84	0.58	0.77	0.6	0.66	0.59	0.95	0.6
	5	0.8	0.62	0.82	0.73	0.71	0.64	0.88	0.51
	6	0.73	0.57	0.77	0.73	0.75	0.61	0.85	0.64
BIC	3	0.96	0.86	0.53	0.65	0.77	0.82	0.95	0.81
	4	0.91	0.87	0.67	0.81	0.84	0.79	0.85	0.78
	5	0.69	0.63	0.63	0.67	0.69	0.67	0.65	0.74
	6	0.67	0.74	0.61	0.6	0.66	0.55	0.59	0.74
COAD	3	0.97	0.82	0.7	0.67	0.75	0.58	0.83	0.82
	4	0.65	0.7	0.74	0.57	0.69	0.53	0.6	0.67
	5	0.8	0.68	0.72	0.59	0.56	0.45	0.96	0.73
	6	0.89	0.69	0.59	0.527	0.43	0.41	0.69	0.65
KRCC	3	0.83	0.77	0.58	0.48	0.65	0.64	0.95	0.63
	4	0.78	0.8	0.65	0.56	0.81	0.68	0.95	0.49
	5	0.55	0.67	0.59	0.46	0.79	0.64	0.78	0.58
	6	0.7	0.59	0.65	0.53	0.75	0.62	0.67	0.68

**Table 3:** Results of autoencoder with data filtered by COX Index.

Dataset	Number of Cluster	Autoencoder Vanilla		Autoencoder Denoising		Autoencoder Sparse		Autoencoder Variational	
		PAM/Spearman	k-Means/ Euclidean	PAM/Spearman	k-Means/Euclidean	PAM/Spearman	k-Means/Euclidean	PAM/Spearman	k-Means/Euclidean
COAD	3	0.0002	0.0027	0.0025	0.0025	0.005	0.005	0.0024	0.0027
	4	0.0081	0.0067	0.0076	0.0076	0.162	0.0072	9 × 10${}^{-5}$	0.012
	5	0.016	0.016	0.0097	0.0097	0.0253	0.0017	0.0032	0.026
	6	0.0323	0.0217	0.0205	0.015	0.0007	0.0082	0.0082	0.051
KRCC	3	4 × 10${}^{-9}$	7 × 10${}^{-8}$	1 × 10${}^{-8}$	8 × 10${}^{-5}$	0.1	1 × 10${}^{-6}$	0.006	0.026
	4	5 × 10${}^{-9}$	3 × 10${}^{-7}$	9 × 10${}^{-12}$	1 × 10${}^{-6}$	0.1	5 × 10${}^{-6}$	0.035	0.039
	5	9 × 10${}^{-11}$	3 × 10${}^{-8}$	1 × 10${}^{-10}$	2 × 10${}^{-8}$	0.5	2 × 10${}^{-5}$	0.1	0.014
	6	3 × 10${}^{-10}$	9 × 10${}^{-7}$	1 × 10${}^{-12}$	6 × 10${}^{-8}$	0.4	3 × 10${}^{-5}$	0.67	0.032
**Silhoutte Index Result**									
COAD	3	0.99	0.91	1	0.85	1	0.9	0.88	0.96
	4	0.95	0.76	0.98	0.76	0.98	0.76	0.85	0.78
	5	0.98	0.67	0.83	0.68	0.82	0.65	0.93	0.78
	6	0.87	0.63	0.87	0.6	0.77	0.63	0.81	0.6
KRCC	3	0.74	0.82	0.77	0.83	0.28	0.1	0.95	0.63
	4	0.68	0.74	0.69	0.8	0.38	0.1	0.95	0.49
	5	0.64	0.71	0.66	0.64	0.48	0.22	0.78	0.58
	6	0.54	0.62	0.75	0.6	0.55	0.26	0.66	0.68

**Table 4:** PCA and SNF Results.

Principal Component Analysis Results							
**Dataset**	**Number** **of Cluster**	**PCA**		**Kernel PCA**		**Sparse PCA**	
		***p*** **-Value**	**Silhoutte** **Index**	***p*** **-Value**	**Silhoutte** **Index**	***p*** **-Value**	**Silhoutte** **Index**
GBM	3	0.542	0.56	0.459	0.23	0.396	0.65
	4	0.514	0.42	0.668	0.31	0.492	0.61
	5	0.989	0.35	0.506	0.5	0.104	0.61
	6	0.731	0.38	0.89	0.5	0.113	0.58
**Similarity Network Fusion Results**							
**Dataset**	**Number** **of Cluster**	***p*** **-Value**			**Silhoutte** **Index**		
GBM	3	2.43 × 10${}^{-5}$			0.46		
	4	0.001			0.47		
	5	3.39 × 10${}^{-5}$			0.47		
	6	1.92 × 10${}^{-5}$			0.46		

## Data Availability

The data and the codes are available online at https://github.com/edianfranklin/autoencoder_for_cancer_subtype (accessed on 17 March 2021).

## Supplementary Materials

The following are available online at https://www.mdpi.com/article/10.3390/cancers13092013/s1, Figure S1: Data results images, Figure S2: GBM Differential Expression Analysis Results.

## Abbreviations

The following abbreviations are used in this manuscript:

TCGA	The Cancer Genome Atlas
SNF	Similarity network fusion
DL	Deep learning
GBM	Glioblastoma multiforme
COAD	Colon Adenocarcinoma
KRCC	Kidney renal clear cell carcinoma
BIC	Breast invasive carcinoma
VAR	Maximum variance
PCA	Principal Component Analysis
PAM	Partitioning around medoids
DE	Differential expression
GO	Gene Ontology
CL1	Cluster 1
GEO	Gene Expression Omnibus
TME	surrounding tumor microenvironmen
